# Does a Loading Dose Improve Outcomes? Colistin Therapy for Urinary Tract Infections Caused by Multidrug-Resistant Gram-Negative Bacteria

**DOI:** 10.3390/antibiotics15080812

**Published:** 2026-08-19

**Authors:** Wasan Katip, Puntapong Taruangsri, Siriporn Okonogi, Shaun Wen Huey Lee, Ajaree Rayanakorn

**Affiliations:** 1 Department of Pharmaceutical Care, Faculty of Pharmacy, Chiang Mai University, Chiang Mai 50200, Thailand; 2 Center of Excellence in Pharmaceutical Nanotechnology, Faculty of Pharmacy, Chiang Mai University, Chiang Mai 50200, Thailand; siriporn.okonogi@cmu.ac.th; 3 Department of Medicine, Nakornping Hospital, Chiang Mai 50180, Thailand; puntapong@yahoo.com; 4 Department of Pharmaceutical Sciences, Faculty of Pharmacy, Chiang Mai University, Chiang Mai 50200, Thailand; 5 School of Pharmacy, Monash University Malaysia, Jalan Lagoon Selatan, Bandar Sunway 47500, Selangor, Malaysia; 6 Department of Pharmacology, Faculty of Medicine, Chiang Mai University, Chiang Mai 50200, Thailand

**Keywords:** colistin, loading dose, urinary tract infection, nephrotoxicity, colistimethate sodium, antimicrobial resistance, multidrug-resistant Gram-negative bacteria, antimicrobial stewardship, SDG 3, good health and well-being

## Abstract

**Background:** Colistin remains an important therapeutic option for complicated urinary tract infections (cUTIs) caused by multidrug-resistant Gram-negative bacteria. A loading dose is often recommended to rapidly achieve steady-state plasma concentrations, but its clinical benefit specifically for cUTIs, where local urinary drug concentrations markedly exceed plasma levels, remains unclear. **Methods:** We conducted a retrospective cohort study of adult patients treated with intravenous colistin for cUTIs at Nakornping Hospital, Chiang Mai, Thailand, between 2015 and 2022. Patients were classified as receiving loading-dose (LD) or non-loading-dose (non-LD) colistin. The primary outcome was 30-day all-cause mortality. Secondary outcomes included clinical response, microbiological response, and nephrotoxicity (defined by RIFLE criteria). Inverse probability weighting (IPW) using the propensity score was applied to adjust for baseline covariates, and Cox proportional hazards regression was used to estimate crude and adjusted hazard ratios (HR) using the non-LD group as the reference. **Results:** Among 123 patients (74 LD, 49 non-LD), unadjusted 30-day all-cause mortality was 35.14% versus 40.82% (*p* = 0.524), clinical response was 75.68% versus 71.43% (*p* = 0.599), and microbiological response was 78.38% versus 65.31% (*p* = 0.109) for LD versus non-LD, respectively. Nephrotoxicity occurred in 48.65% versus 38.78% (*p* = 0.281). After IPW adjustment, no statistically significant differences were observed between the LD and non-LD groups for 30-day all-cause mortality (aHR 0.78, 95% CI 0.43–1.42, *p* = 0.419), clinical response (aOR 1.48, 95% CI 0.54–4.08, *p* = 0.445), or microbiological response (aOR 2.10, 95% CI 0.81–5.48, *p* = 0.128). For the safety outcome, nephrotoxicity was analyzed using the Fine–Gray competing risk model, treating death as a competing event. The IPW-adjusted subdistribution hazard ratio was (asHR) 2.23 (95% CI 0.83–5.95, *p* = 0.110), indicating no statistically significant difference in renal safety between the LD and non-LD groups. **Conclusions:** Loading-dose colistin was not associated with a statistically significant reduction in 30-day all-cause mortality or improvements in clinical response and microbiological eradication in patients with cUTIs. Nephrotoxicity was numerically lower without a loading dose, but the wide confidence intervals in this study of 123 patients precluded a definitive conclusion regarding renal safety. Omitting the loading dose may be a reasonable option for localized urinary infections, particularly in patients at high risk for acute kidney injury, pending confirmation in larger prospective studies.

## 1. Introduction

Urinary tract infections (UTIs) caused by antibiotic-resistant Gram-negative bacteria have become an increasingly pressing clinical problem, largely because the therapeutic options available to treat them are shrinking. Enterobacteriaceae remain the most frequent causative organisms in both community-acquired and hospital-acquired UTIs. However, these organisms are increasingly acquiring resistance mechanisms, including extended-spectrum beta-lactamases (ESBL), AmpC beta-lactamases, and carbapenemases, which substantially limit the antibiotics that remain effective for treatment [1,2]. For UTIs caused by carbapenem-resistant Enterobacteriaceae, treatment options are limited to a relatively small group of agents, including ceftazidime–avibactam, fosfomycin, aztreonam, aminoglycosides, tigecycline (although its clinical utility in UTIs is limited by low urinary excretion), and the polymyxins, namely, colistin and polymyxin B [1,3,4]. Similarly, for UTIs caused by multidrug-resistant Pseudomonas species, colistin is recognized alongside ceftazidime–avibactam, ceftolozane–tazobactam, and aminoglycosides as one of the few remaining active agents once first-line beta-lactams and fluoroquinolones can no longer be relied upon [1,4].

Within this shrinking therapeutic landscape, colistin has re-emerged as an important option because of its reliable in vitro activity against extensively drug-resistant Gram-negative organisms, including *Acinetobacter baumannii* and *Pseudomonas aeruginosa*. A meta-analysis comparing colistin with tigecycline for the treatment of multidrug-resistant and extensively drug-resistant Gram-negative infections confirmed that colistin remains an effective and commonly used option in this setting, and it continues to be used as salvage or rescue therapy in patients infected with carbapenem-resistant *A. baumannii* who have few remaining treatment alternatives [5]. Despite this clinical reliance on colistin, an optimal dosing strategy for its inactive prodrug, colistimethate sodium (CMS), has still not been firmly established. This uncertainty is compounded by pharmacokinetic data showing that colistin has a comparatively long elimination half-life. A population pharmacokinetic analysis of a conventional CMS dosing regimen of 100 mg colistin base activity (3 million units) administered every 8 h found that, as a direct consequence of this long half-life, plasma colistin concentrations remained inadequate for approximately the first 12 to 48 h after treatment initiation [6]. This finding has been used to justify the administration of a loading dose at the start of therapy, on the rationale that a larger initial dose can accelerate the attainment of steady-state plasma concentrations and thereby avoid a period of subtherapeutic systemic exposure early in treatment.

However, whether this pharmacokinetic rationale for a loading dose, developed primarily with systemic and invasive infections, translates into meaningful clinical benefit for complicated urinary tract infections (cUTIs) remains uncertain. Sorlí et al. [7] emphasized that adequate dosing is essential to ensure the efficacy of colistin therapy. However, nephrotoxicity remained common in their cohort, which the authors attributed to the substantial renal excretion of colistimethate sodium (CMS) and its local urinary conversion to active colistin. These findings highlight the complex balance between achieving adequate systemic exposure for antimicrobial efficacy and minimizing the risk of renal toxicity. These findings are biologically plausible because urinary CMS and colistin concentrations substantially exceed plasma concentrations, with CMS being extensively excreted by the kidneys and converted to active colistin within the urinary tract. Consequently, antimicrobial efficacy in cUTIs may depend more on high local urinary drug exposure than on rapid attainment of systemic plasma concentrations through a loading dose [7].

Taken together, this body of pharmacokinetic findings suggests that a loading dose may provide limited additional benefit for urinary tract infections (UTIs), as the urinary tract is exposed to high local colistin concentrations largely independent of the rate at which systemic steady-state concentrations are achieved. However, this hypothesis is based primarily on pharmacokinetic principles rather than direct clinical evidence, and comparative studies evaluating the impact of a loading dose in patients with UTIs treated with colistin remain scarce. To address this knowledge gap, we compared clinical outcomes between loading-dose and non-loading-dose colistin in patients with UTIs. The primary outcome was 30-day all-cause mortality, while secondary outcomes included clinical response, microbiological response, and nephrotoxicity.

## 2. Results

A total of 123 patients treated with intravenous colistin were included in the analysis, of whom 74 (60.2%) received a loading dose and 49 (39.8%) did not. The baseline demographic and clinical characteristics of the two groups are summarized in Table 1.

The proportion of male patients was numerically higher in the loading-dose group than in the non-loading-dose group (39.19% vs. 24.49%), although this difference did not reach statistical significance (*p* = 0.090). Patients in the non-loading-dose group tended to be older than those in the loading-dose group (mean age 66.97 ± 15.97 years vs. 61.85 ± 17.24 years), but this difference was also not statistically significant (*p* = 0.099). The duration of colistin treatment was comparable between the two groups (8.98 ± 5.26 days in the loading-dose group vs. 8.00 ± 4.49 days in the non-loading-dose group; *p* = 0.284).

Absolute dosing data are summarized in Table 2. Colistin was administered intravenously every 12 h (q12h) in all patients in both groups. All patients in the loading-dose group received a uniform initial dose of 300 mg colistin base activity (CBA). In the non-loading-dose group, the initial dose was individualized (range 100–250 mg, most commonly 150 mg). Median total cumulative colistin exposure was significantly higher in the loading-dose group than in the non-loading-dose group (1925 mg, IQR 1143–3000, vs. 1210 mg, IQR 675–2025; *p* < 0.001), as was the median average daily dose (283 mg/day, IQR 175–333, vs. 150 mg/day, IQR 110–210; *p* < 0.001), despite similar treatment duration between groups.

With respect to underlying comorbidities, the prevalence of chronic obstructive pulmonary disease, malignancy, cardiovascular disease, hypertension, and diabetes mellitus did not differ significantly between groups (all *p* > 0.05). However, patients in the non-loading-dose group had a significantly higher prevalence of chronic kidney disease compared with the loading-dose group (42.86% vs. 21.62%, *p* = 0.012). A trend toward a higher prevalence of chronic liver disease was also observed in the non-loading-dose group, although this did not reach statistical significance (10.20% vs. 4.05%, *p* = 0.176).

The proportion of patients admitted to the intensive care unit (40.54% vs. 40.82%) and the incidence of septic shock (47.30% vs. 38.78%) were similar between the loading-dose and non-loading-dose groups (*p* = 0.976 and *p* = 0.351, respectively). Notably, the mean Charlson comorbidity index score was significantly higher in the non-loading-dose group than in the loading-dose group (3.40 ± 2.47 vs. 2.35 ± 2.05, *p* = 0.011), indicating a greater overall burden of comorbidity among patients who did not receive a loading dose. Consistent with this, baseline serum creatinine (SCr) was also significantly higher in the non-loading-dose group compared with the loading-dose group (1.88 ± 1.62 mg/dL vs. 1.23 ± 1.34 mg/dL, *p* = 0.022), suggesting poorer baseline renal function in this group.

Given the high overall severity of illness in this cohort, we additionally examined markers relevant to the potential for extra-urinary infection. Mechanical ventilation at the time of colistin initiation was common overall (71/123 patients, 57.7%; 59.5% in the loading-dose group vs. 55.1% in the non-loading-dose group). A concurrent extra-urinary diagnosis, coded alongside UTI in the medical record, was present in 25/123 patients (20.3%; 21.6% in the loading-dose group vs. 18.4% in the non-loading-dose group), including bacteremia/septicemia, sepsis, hospital-acquired pneumonia, tissue infection, and infected peritoneal dialysis catheter. Catheterization status and blood culture results were not systematically recorded in this retrospective dataset and could not be reported; this is addressed further in the Section 3.

Regarding concomitant use of nephrotoxic medications, no statistically significant differences were observed between groups for aminoglycosides, diuretics, amphotericin B, or vasopressors (all *p* > 0.05). Although vancomycin use was more frequent in the loading-dose group compared with the non-loading-dose group (52.70% vs. 38.78%), this difference did not reach statistical significance (*p* = 0.130).

The length of hospital stay was similar between the two groups (40.28 ± 34.07 days in the loading-dose group vs. 37.79 ± 26.02 days in the non-loading-dose group, *p* = 0.665). With regard to the causative organisms, *Acinetobacter baumannii* was the most commonly isolated pathogen in both groups (68.92% in the loading-dose group vs. 61.22% in the non-loading-dose group), followed by *Pseudomonas aeruginosa*, *Escherichia coli*, and *Klebsiella pneumoniae*. The distribution of causative pathogens did not differ significantly between groups (all *p* > 0.05).

Overall, the two treatment groups were largely comparable in terms of baseline demographic characteristics, comorbidities, severity of illness, concomitant nephrotoxic drug use, and causative organisms. However, patients who did not receive a loading dose had a significantly higher burden of comorbidity (as reflected by the Charlson score) and poorer baseline renal function (as reflected by baseline serum creatinine), along with a higher prevalence of chronic kidney disease, compared with those who received a loading dose.

### 2.1. Primary and Secondary Outcomes

**Figure 1:**
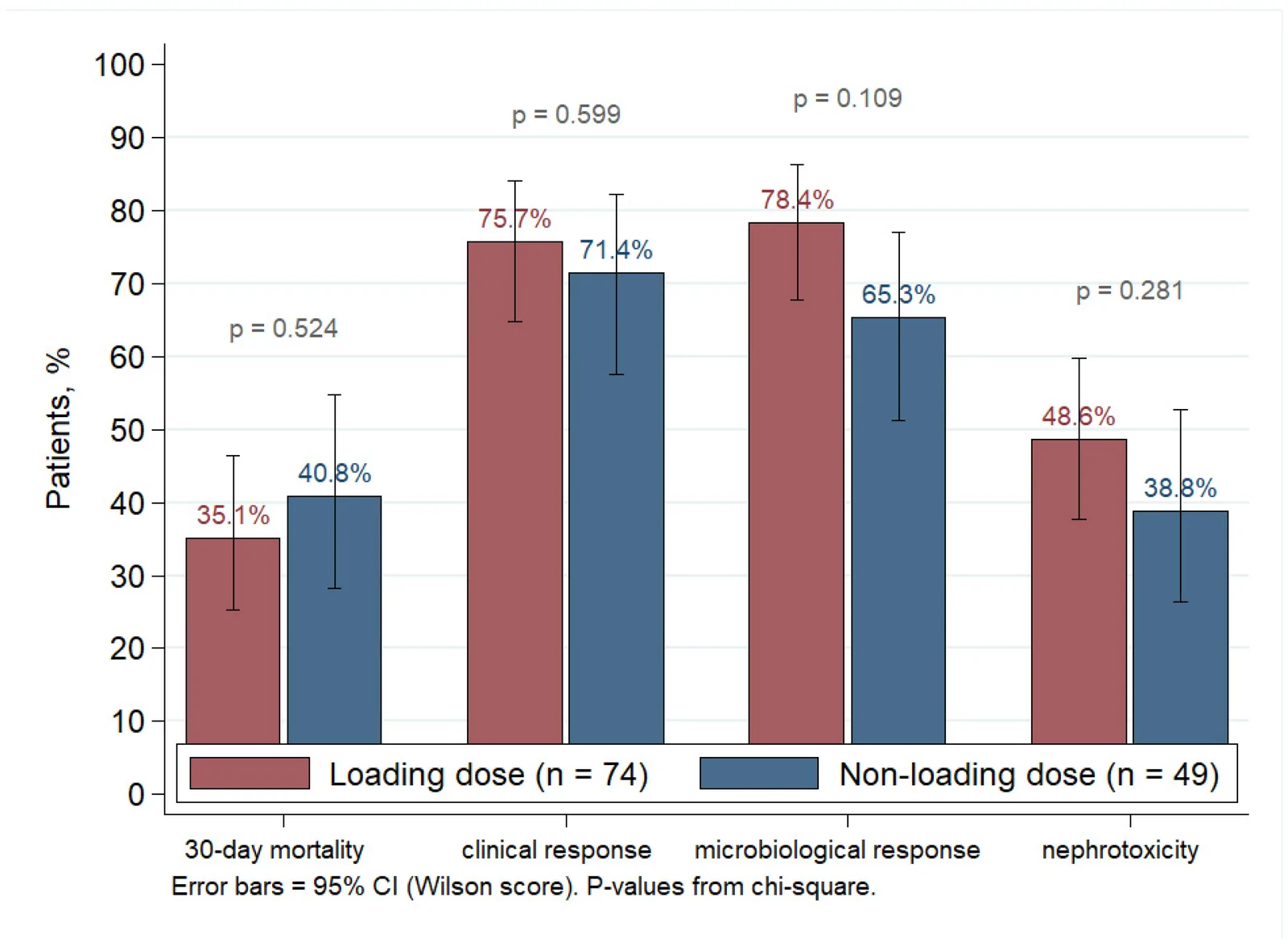
Comparison of primary and secondary outcomes between loading-dose and non-loading-dose colistin groups, with 95% confidence intervals (Wilson score method). Error bars represent 95% CI for each proportion. *p*-values from chi-square.

The primary and secondary outcomes for patients who received loading-dose colistin compared with those who received non-loading-dose colistin are summarized in Table 3 and visualized with their corresponding 95% confidence intervals in Figure 1. Thirty-day all-cause mortality was numerically lower in the loading-dose group than in the non-loading-dose group (35.14% vs. 40.82%), but this difference was not statistically significant (*p* = 0.524) (Table 3).

Among the secondary outcomes shown in Table 3, clinical response was achieved in 75.68% of patients in the loading-dose group and 71.43% in the non-loading-dose group, with no statistically significant difference between groups (*p* = 0.599). Similarly, microbiological response was higher in the loading-dose group than in the non-loading-dose group (78.38% vs. 65.31%). However, the difference was not statistically significant (*p* = 0.109) (Table 3).

Regarding safety (Table 3), nephrotoxicity occurred in 48.65% of patients in the loading-dose group and 38.78% in the non-loading-dose group, with no statistically significant difference between groups (*p* = 0.281).

### 2.2. Cox Regression Analysis

To further evaluate the association between loading-dose colistin therapy and clinical outcomes, regression analyses were performed with inverse probability weighting (IPW) based on the propensity score to adjust for baseline covariates, including age, gender, liver cirrhosis, chronic kidney disease, Charlson comorbidity index, baseline serum creatinine, and concomitant vancomycin use. The results are presented in Table 4, with the non-loading-dose group as the reference.

**Figure 2:**
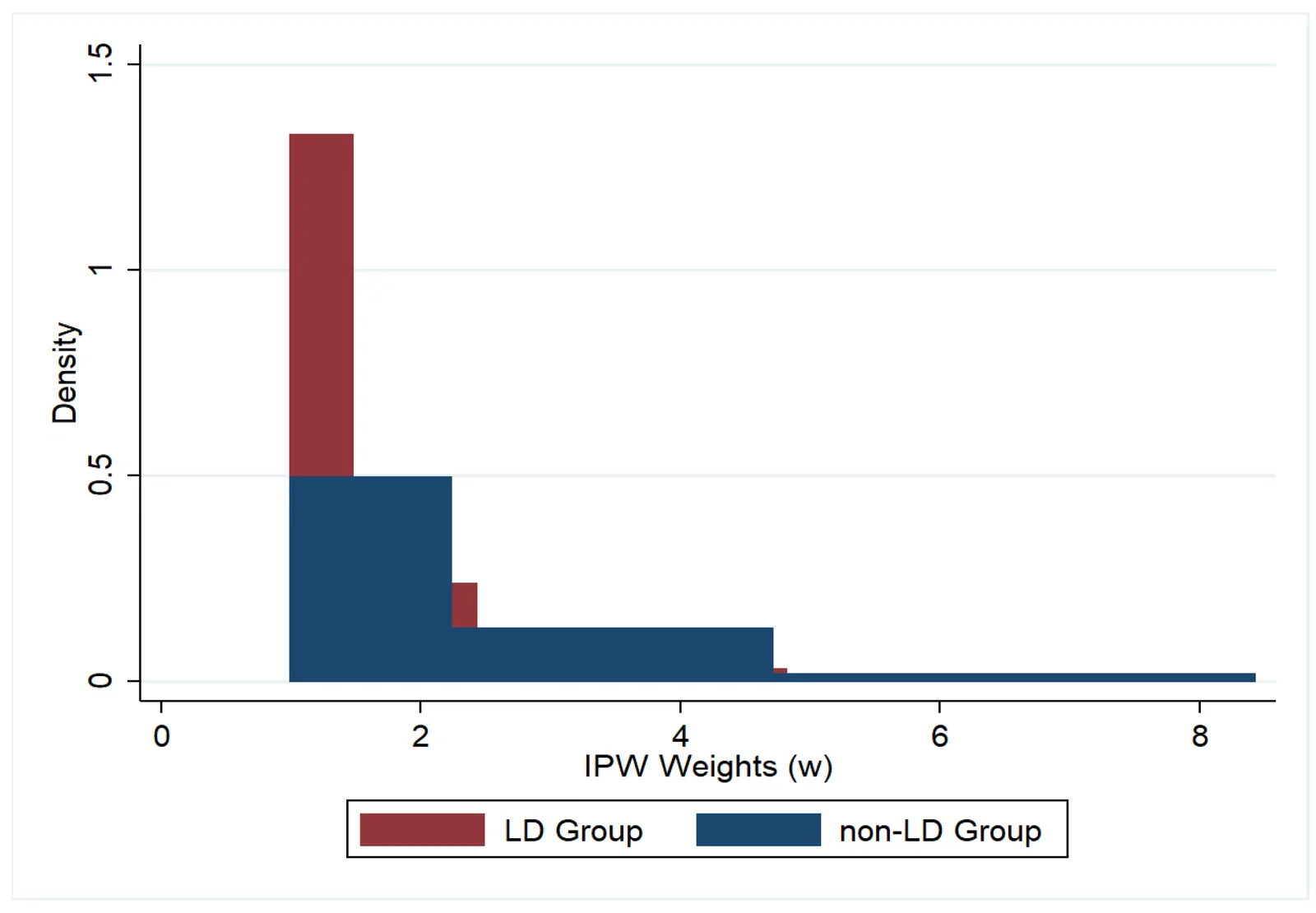
Distribution of inverse probability weighting (IPW) weights for the loading-dose (LD) and non-LD groups. The histogram illustrates a stable weighting profile, with the majority of weights clustered between 1 and 4 and no extreme outliers (maximum weight approximately 8). This distribution ensures the statistical stability of the propensity-weighted survival model and justifies the shift in the adjusted hazard ratio.

**Figure 3:**
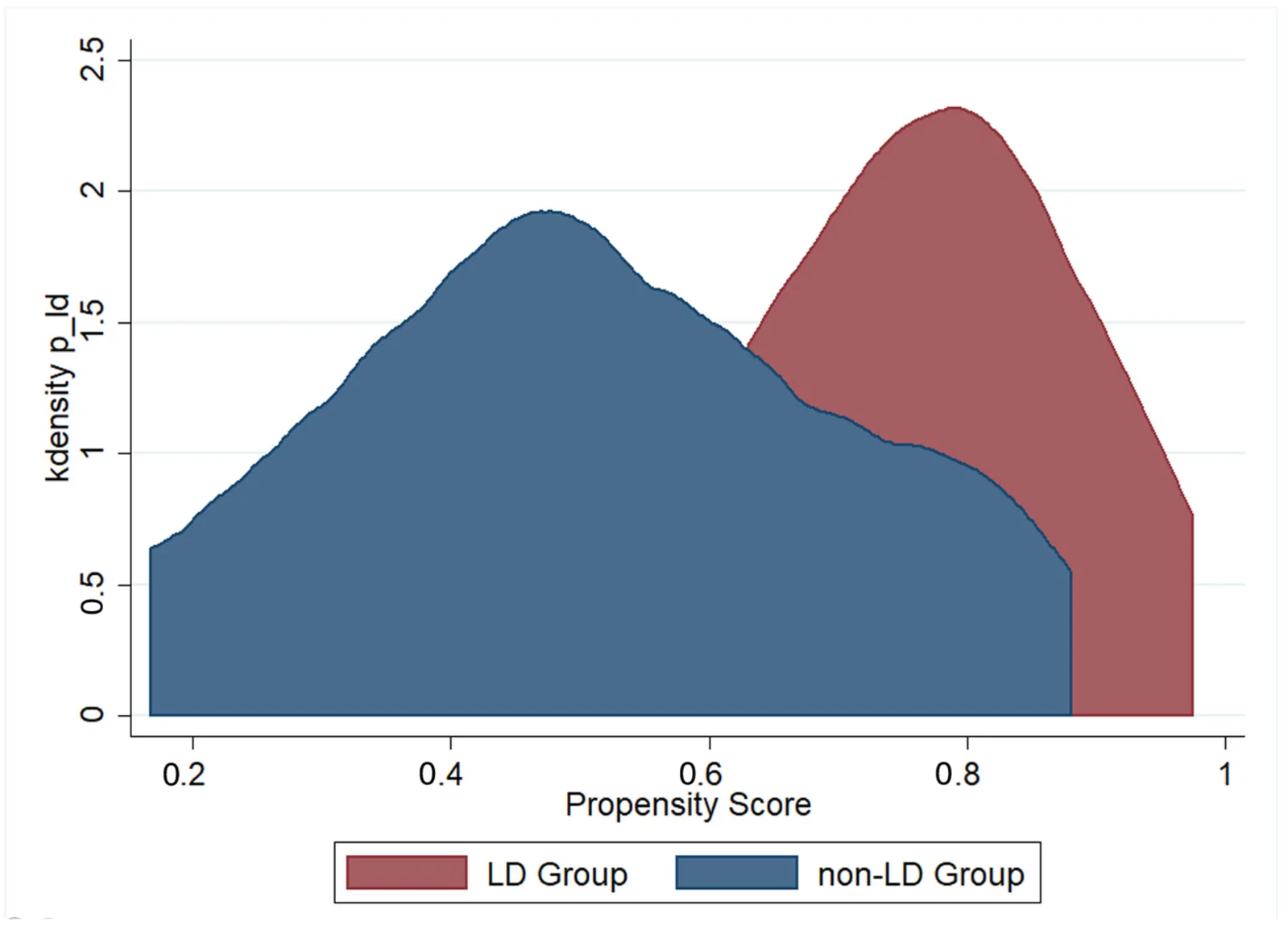
Propensity score overlap plot demonstrating the common support between the loading-dose (LD) and non-LD groups. The kernel density curves show **substantial overlap** across the propensity score spectrum, particularly within the 0.3 to 0.8 range. This visual evidence confirms that patients in both cohorts shared similar baseline characteristics (calculated from variables: age, gender, baseline serum creatinine, Charlson comorbidity index, chronic kidney disease, chronic liver disease, and the concomitant use of vancomycin), validating the use of propensity score weighting for this study.

To ensure the statistical validity of this propensity-weighted survival model, we evaluated the distribution of the generated IPW weights (Figure 2). The histogram reveals a highly stable weighting profile, with the majority of patient weights clustered tightly between 1 and 4 and no extreme outliers (maximum weight approximately 8). Furthermore, the propensity score overlap plot demonstrates substantial common support and an excellent baseline balance between the loading-dose and non-loading-dose groups, particularly within the 0.3 to 0.8 range (Figure 3).

**Figure 4:**
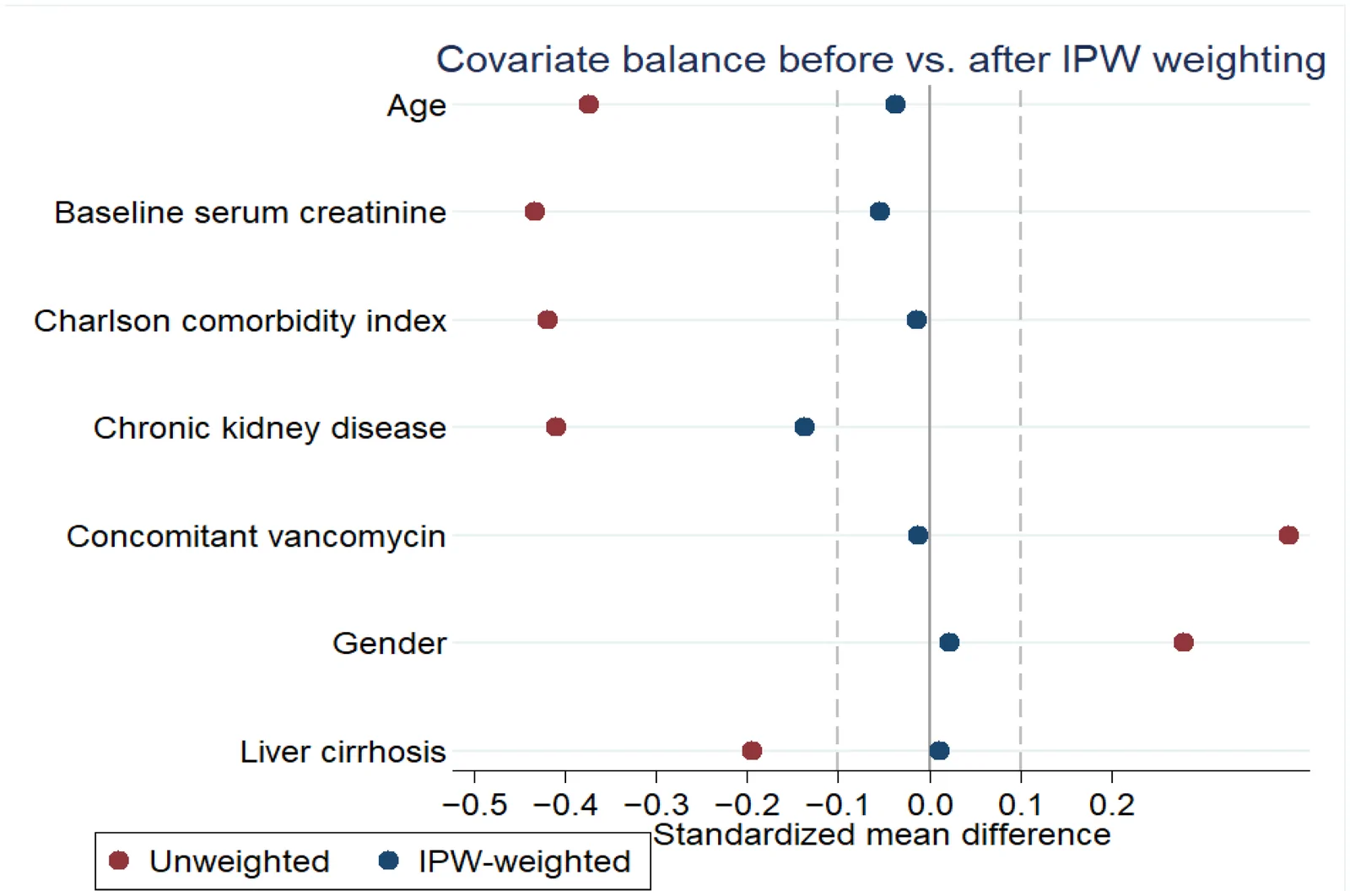
Covariate balance (standardized mean difference) before and after IPW weighting. The solid grey vertical line indicates a standardized mean difference (SMD) of 0 (no imbalance), and the dashed grey vertical lines indicate the conventional threshold of ±0.10, below which covariate balance is considered adequate.

The successful reduction of baseline imbalances is confirmed by the covariate balance plot (Figure 4), which shows that after IPW weighting, the standardized mean differences (SMD) for all adjusted baseline variables shifted well below the conventional threshold of ±0.10.

In the crude (unadjusted) analyses shown in Table 4, loading-dose colistin was not significantly associated with 30-day all-cause mortality (HR 0.76, 95% CI 0.43–1.38; *p* = 0.370), clinical response (OR 1.24, 95% CI 0.55–2.81; *p* = 0.599), microbiological response (OR 1.81, 95% CI 0.74–4.42; *p* = 0.118), or nephrotoxicity (sHR 1.57, 95% CI 0.65–3.83; *p* = 0.319) compared with the non-loading-dose group.

After IPW adjustment, the associations remained non-significant across all outcomes. The IPW-adjusted hazard ratio for 30-day all-cause mortality was 0.78 (95% CI 0.43–1.42; *p* = 0.419). For secondary outcomes, the IPW-adjusted odds ratio for clinical response was 1.48 (95% CI 0.54–4.08; *p* = 0.445), and that for microbiological response was 2.10 (95% CI 0.81–5.48; *p* = 0.128). For safety, nephrotoxicity was analyzed using a Fine–Gray competing risk approach treating death as a competing event. The IPW-adjusted subdistribution hazard ratio was 2.23 (95% CI 0.83–5.95; *p* = 0.110).

Overall, the results in Table 3 and Table 4 indicate that the loading-dose regimen was not associated with statistically significant improvements in 30-day all-cause mortality, clinical response, or microbiological response compared with non-loading-dose therapy. Although point estimates for nephrotoxicity were numerically higher in the loading-dose group in both crude and adjusted analyses, these differences did not reach statistical significance.

We assessed covariate balance using standardized mean differences (SMDs) for seven baseline covariates before and after inverse probability weighting (IPW). Before weighting, all covariates exceeded the conventional imbalance threshold (|SMD| > 0.1). After IPW, covariate balance improved substantially, with only chronic kidney disease remaining slightly imbalanced (SMD = −0.136), and no covariates exceeding |SMD| > 0.2 (Figure 4).

**Figure 5:**
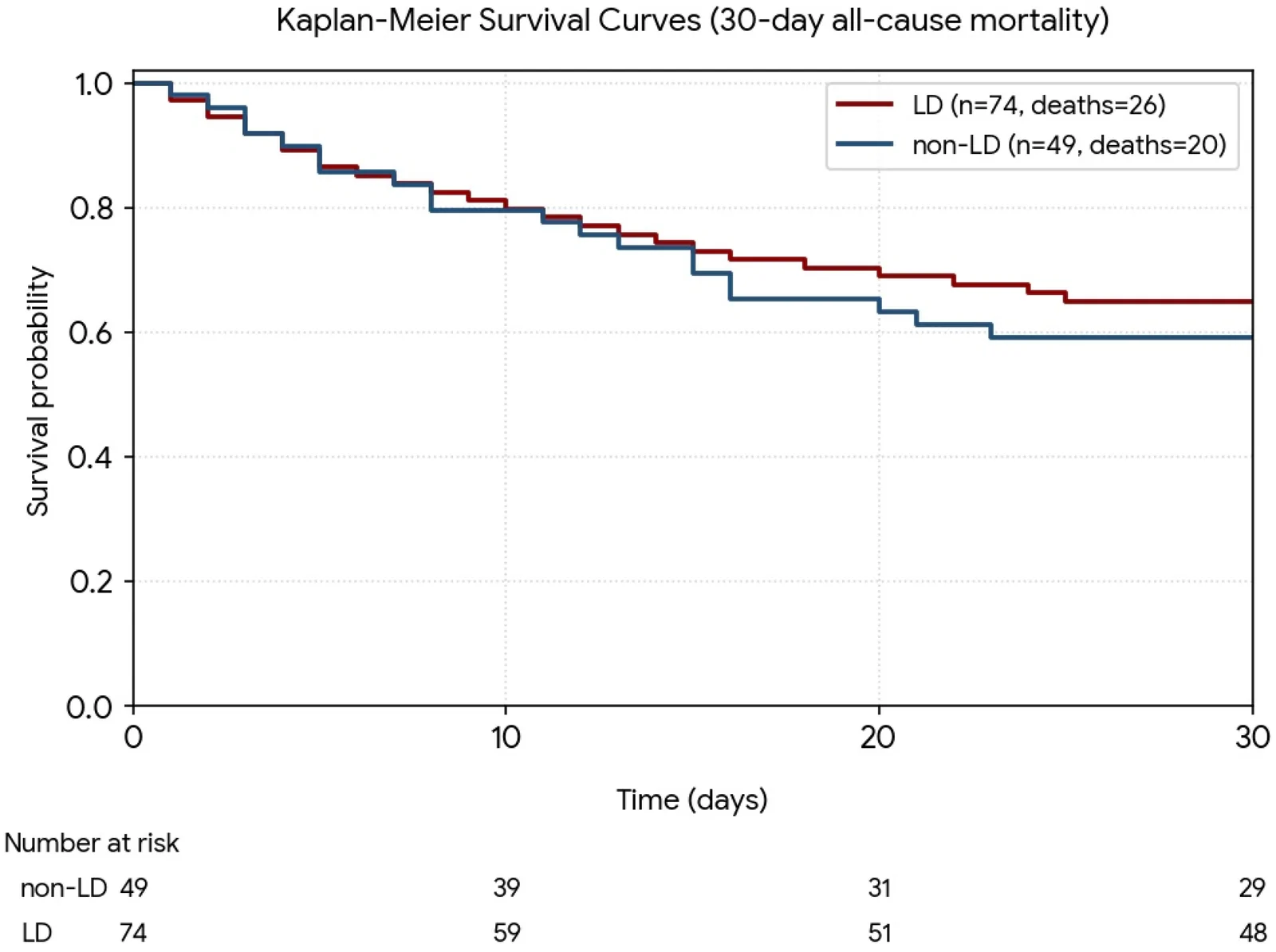
Kaplan–Meier survival curves for 30-day all-cause mortality comparing loading-dose (LD) colistin versus non-loading-dose colistin therapy.

The Kaplan–Meier survival curves are shown in Figure 5. The estimated 30-day all-cause mortality was numerically higher in patients who received loading-dose (LD) colistin than in those who received non-LD colistin. However, the curves showed substantial overlap during follow-up. This finding was consistent with Cox regression results, which showed no statistically significant difference in 30-day all-cause mortality between groups (crude HR 0.76, 95% CI 0.43–1.38; *p* = 0.370; IPW-adjusted HR 0.78, 95% CI 0.43–1.42; *p* = 0.419; Table 4).

## 3. Discussion

In this study, we compared clinical, microbiological, and safety outcomes between patients who received a loading dose of colistin and those who did not. Despite baseline imbalances favoring the loading-dose group, namely, a lower prevalence of chronic kidney disease, a lower Charlson comorbidity index, and lower baseline serum creatinine (Table 1), neither the crude nor the inverse probability weighting (IPW)-adjusted analyses demonstrated a statistically significant benefit of loading dose colistin in reducing 30-day all-cause mortality or improving clinical and microbiological responses (Table 3 and Table 4). Similarly, no significant difference in nephrotoxicity was observed between groups, although the adjusted hazard ratio for nephrotoxicity trended higher in the loading-dose group (asHR 2.23, 95% CI 0.83 to 5.95). These findings raise important questions about the actual clinical value of a loading dose strategy for colistin in the treatment of complicated urinary tract infections (cUTIs), and they align in several respects, though diverge in others, from the previously published literature.

### 3.1. Comparison with Existing Evidence on Colistin Dosing and Clinical Outcomes in UTI

Our finding that a substantial proportion of patients achieved clinical cure regardless of loading dose status is consistent with prior observations that plasma pharmacokinetic/pharmacodynamic (PK/PD) targets for colistin may not fully predict clinical success in urinary tract infections. Sorlí and colleagues, in a prospective cohort of patients with UTI caused by extensively drug-resistant (XDR) *Pseudomonas aeruginosa*, reported a clinical cure rate of nearly 94% despite the fact that only about one-third of patients achieved the recommended plasma AUC/MIC target of 60 mg·h/L or greater, and fewer than 10% reached the proposed therapeutic plasma concentration of 2.5 mg/L [7]. The authors proposed that this apparent disconnect between plasma exposure and clinical outcome reflects the unique pharmacology of colistimethate sodium (CMS). Because the inactive prodrug CMS is extensively excreted via the kidneys and subsequently converted to active colistin within the urinary tract itself, local urinary concentrations of the active moiety can far exceed plasma levels. This makes plasma-based PK/PD targets, and by extension dosing strategies aimed at rapidly achieving steady-state plasma concentrations such as a loading dose, less relevant for cUTIs than for invasive infections at other systemic sites [7].

Our results, showing no added clinical or microbiological benefit from a loading dose, are compatible with this interpretation and are further corroborated by recent real-world data. Aldairem et al. [8], in a cohort study investigating colistin therapy for UTIs due to multidrug-resistant bacteria, demonstrated favorable clinical and microbiological eradication rates even though a loading dose was omitted in the vast majority (over 86%) of patients. If the therapeutic effect of colistin in UTI is driven predominantly by high local urinary concentrations generated progressively through regular dosing rather than by rapid attainment of steady-state plasma levels, then omitting a loading dose may not meaningfully compromise treatment efficacy [8,9].

This interpretation is further supported by Karakkattu et al. [10], who reported an 80% favorable clinical outcome among 80 patients treated with colistin for multidrug-resistant UTI, even though only 10 of these patients received a loading dose. The authors specifically noted that clinicians in their cohort were frequently reluctant to administer a loading dose because of concerns about precipitating nephrotoxicity, despite loading-dose strategies having been proposed to more rapidly achieve steady-state drug concentrations [10]. This clinical hesitancy mirrors the pattern in our own cohort, where the non-loading-dose group was numerically smaller (*n* = 49 vs. *n* = 74) and carried a heavier burden of comorbidity and renal impairment at baseline, suggesting that in real-world practice, loading doses may be preferentially withheld in patients perceived to be at higher baseline risk of renal injury. This prescribing pattern represents a classic example of confounding by indication, which our propensity score weighting (IPW) design specifically sought to minimize and adjust for.

### 3.2. Urinary Versus Plasma Colistin Exposure as an Explanation for the Lack of Loading Dose Benefit

A plausible pharmacological explanation for why loading dose status did not significantly influence clinical or microbiological outcomes in our cohort relates to the disproportionate distribution of colistin between plasma and urine. Sorlí et al. [7] demonstrated that, in their cohort of 33 patients with UTI caused by extensively drug-resistant *Pseudomonas aeruginosa*, the mean steady-state plasma colistin concentration (Css) was only 1.19 mg/L, with wide individual variability (range 0.21 to 5.20 mg/L). Only 9.1% of patients achieved a plasma concentration above the proposed therapeutic target of 2.5 mg/L, and only 33.3% attained the optimal plasma AUC/MIC index of 60 mg·h/L or greater. Despite these largely suboptimal plasma exposures, clinical cure was achieved in 93.9% of patients overall and in 89.5% of those treated with colistin monotherapy, and microbiological eradication occurred in 82.6% of patients. The authors concluded that this apparent mismatch between low plasma drug exposure and high clinical success is best explained by the fact that colistimethate sodium, the inactive prodrug of colistin, is extensively renally excreted and converted to its active form largely within the urinary tract itself, resulting in urinary colistin concentrations that likely far exceed those measured in plasma [7]. Indeed, the majority of patients in that study (66.7%) had plasma concentrations above the MIC of the isolated organism even without reaching formal PK/PD targets, and the authors explicitly noted that measuring urinary rather than plasma colistin concentrations may be a more clinically meaningful predictor of treatment success in UTI [7].

This pharmacological principle is directly relevant to the interpretation of our own findings. A loading dose is designed primarily to shorten the time required to reach steady-state plasma drug concentrations, a strategy that is most clinically important when the therapeutic target of interest is systemic (plasma) drug exposure, such as in bacteremia, pneumonia, or other invasive infections where colistin must act at a tissue site supplied only through the bloodstream. In contrast, in localized infections such as cUTIs, the bladder and urinary tract are exposed to colistin primarily through renal excretion of CMS and its subsequent conversion to active colistin, a process that occurs with each administered dose regardless of whether a loading dose is used. Because urinary colistin concentrations in UTI are governed more by cumulative renal excretion over the course of therapy than by the rate at which plasma steady state is achieved, the theoretical advantage of a loading dose, namely, faster attainment of adequate systemic exposure, may simply be less consequential for infections confined to the urinary tract [9].

This interpretation is also consistent with previous clinical evidence highlighting the need to balance adequate colistin exposure against the risk of nephrotoxicity. Sorlí et al. [7] reported a substantial incidence of nephrotoxicity despite the importance of maintaining adequate dosing for treatment efficacy. In this context, our finding that omission of the initial loading dose was not associated with reduced treatment efficacy, together with a favorable trend toward lower nephrotoxicity, suggests that avoiding an early increase in systemic exposure may offer a potential renal-sparing advantage in patients with cUTIs. However, this finding should be interpreted cautiously and warrants confirmation in prospective studies specifically designed to evaluate the efficacy and safety of loading-dose strategies.

This provides a coherent explanation for why we observed no significant difference in 30-day all-cause mortality, clinical response, or microbiological response between the loading-dose and non-loading-dose groups in our cohort (Table 3 and Table 4). This pharmacological reasoning is further reinforced by global consensus data indicating that colistin’s narrow therapeutic index (2–4 mg/L) tightly overlaps with concentrations that induce renal harm, meaning any unnecessary systemic loading could safely be omitted if the target is purely urinary. This is highly consistent with the trend toward a higher adjusted hazard ratio for nephrotoxicity observed in our loading-dose group (asHR: 2.23). This hazard profile aligns with recent findings by Katip et al. (2022) [11], who demonstrated in a multivariate Cox regression that colistin loading doses were independently associated with a statistically significant increase in the hazard of developing acute kidney injury (aHR 1.57, 95% CI 1.14–2.17). Although our study and that of Katip et al. [11] were conducted in the same province with partially overlapping enrollment periods, they were performed at different institutions, Nakornping Hospital and Chiang Mai University Hospital, respectively, with no shared patient population. Taken together, if urinary drug concentrations rather than plasma steady state are the principal determinant of bacterial eradication in UTI, omitting a loading dose may reduce early excessive systemic exposure that contributes to nephrotoxicity without compromising clinical efficacy [8,9].

### 3.3. Relevance of Intravesical and Local Antibiotic Delivery Data

Although conducted with a different antibiotic class and evaluated within an animal rather than a clinical model, the recent experimental work by Yüksel et al. [12] offers a useful conceptual parallel to our observations. In their rat model of cystitis induced by an extensively drug-resistant (XDR) *Acinetobacter baumannii* strain, intravesical tigecycline achieved significant microbiological clearance only after a full five-day treatment course, with no significant differences in bacterial eradication detected earlier in therapy. Furthermore, higher local drug exposure in their study was associated with pronounced tissue inflammation and genotoxic potential in bladder tissues independent of the administered dose. Furthermore, the authors highlighted previous experimental data from Volkow-Fernández et al., which demonstrated that locally delivered colistin achieved superior bladder clearance compared to conventional systemic administration [12,13].

While these experimental models examined local bladder instillation rather than systemic intravenous administration, the broader underlying principle remains highly applicable to our study. Antimicrobial efficacy within the urinary tract appears to depend more on sustained, progressive local drug exposure over time than on rapidly achieved peak concentrations. These findings provide indirect support for our observation that a systemic loading dose, intended to accelerate attainment of therapeutic plasma concentrations, conferred no measurable benefit for clinical or microbiological outcomes in patients with cUTIs. At the same time, the dose-dependent inflammation and safety warnings observed in the animal models serve as a critical reminder that higher drug exposure, even when localized, carries inherent toxicological risks. This concept closely echoes the trend toward increased nephrotoxicity observed in our loading-dose group (asHR: 2.23), suggesting that an initial systemic surge might contribute to safety concerns, although our study was not powered to detect a statistically significant difference [12,13,14].

### 3.4. Nephrotoxicity Findings in Context

The overall nephrotoxicity rates observed in our cohort (48.65% in the loading-dose group and 38.78% in the non-loading-dose group) were numerically higher than those reported in some earlier UTI-specific colistin cohorts. Karakkattu et al. [10] reported nephrotoxicity in 36.3% of patients overall using similar biochemical criteria, while Sorlí et al. [7] reported acute kidney injury at the end of treatment in 30.3% of patients using RIFLE criteria. Differences in nephrotoxicity definitions, patient populations (including baseline renal function and comorbidity burden), and concomitant nephrotoxic drug exposure likely account for these discrepancies.

Our study population represented a highly critically ill cohort, with nearly 40% to 47% of patients presenting with septic shock and over 40% requiring intensive care unit admission. It is well established that underlying critical illness, hemodynamic instability requiring vasopressors, and advanced age exponentially amplify the baseline risk for drug-induced renal injuries [15,16]. Furthermore, the high rate of concomitant vancomycin use (38.8% to 52.7%) in our patients may have acted synergistically with colistin to potentiate renal tubular injury. This synergistic toxicity aligns with large-scale clinical database evidence indicating that combination regimens involving polymyxins and glycopeptides significantly accelerate acute kidney injury (AKI) risk compared to single-agent treatments due to overlapping mechanisms of proximal tubular necrosis [17,18].

Despite the high baseline rate of renal impairment, our IPW-adjusted analysis showed a statistically non-significant numerical increase in the risk of nephrotoxicity in the loading-dose group (asHR 2.23, 95% CI 0.83 to 5.95). This hazard profile is strongly supported by recent systematic evidence. Eljaaly et al. [19] conducted a meta-analysis of five randomized controlled trials (377 patients), in which colistin was used predominantly for pneumonia in intensive care units and compared with beta-lactam-based regimens. Colistimethate sodium was dosed at 9 million units per day (300 mg per day colistin base activity), and a loading dose was administered in four of the five trials. The pooled nephrotoxicity incidence among patients receiving colistin was 36.2% (95% CI 23.3% to 51.3%). Because the analysis compared colistin with non-polymyxin comparators rather than loading dose versus non-loading-dose colistin, it cannot isolate the contribution of a loading dose to nephrotoxicity risk. However, it provides a useful benchmark for the overall frequency of nephrotoxicity reported with colistin in randomized trials. In our cohort, the nephrotoxicity rate in the loading-dose group (48.65%) fell within this reported range [19]. Additionally, a real-world cohort study by Ozel et al. [20] identified elevated systemic colistin concentrations (plasma levels ≥3.5 mg/L) during the first week of therapy—a direct pharmacodynamic consequence of an initial loading dose—as an independent predictor of severe colistin-associated AKI [20]. This suggests that while a loading dose may not compromise or improve clinical cure in cUTIs, it may add an unnecessary, dose-limiting systemic toxic burden to already vulnerable kidneys [20].

Given the sample size of 123 patients and the wide confidence intervals (0.83 to 5.95) for nephrotoxicity, this study could not definitively distinguish between benefit and harm in either direction. While no significant clinical advantage was observed with a loading dose, the lack of statistical significance in safety outcomes means we cannot conclusively rule out or confirm an increased risk of renal injury.

It should also be noted that loading-dose status in our cohort was not independent of maintenance dosing intensity. Median cumulative colistin exposure (1925 mg vs. 1210 mg) and average daily dose (283 mg/day vs. 150 mg/day) were both significantly higher in the loading-dose group than in the non-loading group (both *p* < 0.001), despite similar treatment duration. Because the non-loading group also had a higher baseline prevalence of chronic kidney disease and higher baseline creatinine, lower daily/cumulative dosing in this group likely reflects, at least in part, empirical renal-based dose reduction by treating physicians rather than the absence of a loading bolus per se. Consequently, the numerically lower nephrotoxicity observed in the non-loading group cannot be fully attributed to the omission of a loading dose alone and may partly reflect lower overall drug exposure.

### 3.5. Positioning Colistin Dosing Within Current Treatment Guidance

The 2019 international consensus guidelines on the optimal use of the polymyxins [21] note that the impact of a loading dose on the risk of acute kidney injury remains unclear and recommend that its therapeutic benefit, particularly the need for timely and effective plasma concentrations in critically ill patients, may justify the associated renal risk, while also calling for additional data on the safety and efficacy of loading doses. The rationale for loading is therefore primarily related to the need for prompt attainment of adequate systemic exposure, which may be particularly important in invasive infections such as bacteremia or severe sepsis. In contrast, for urinary tract infections, the pharmacological rationale may differ because CMS undergoes substantial renal elimination and conversion to active colistin within the urinary tract. The clinical benefit of rapidly increasing systemic concentrations may consequently be less pronounced when urinary exposure is an important determinant of treatment efficacy.

This distinction is supported by the 2024 Infectious Diseases Society of America (IDSA) [4] guidance, which identifies colistin, but not polymyxin B, as an alternative agent for uncomplicated cystitis caused by carbapenem-resistant Enterobacterales and difficult-to-treat resistant *Pseudomonas aeruginosa*, owing in part to the conversion of colistin to its active form within the urinary tract. The guidance also emphasizes the nephrotoxicity associated with polymyxins. Importantly, however, these recommendations concern uncomplicated cystitis and should not be interpreted as a specific recommendation for non-loading-dose regimens in cUTI. Rather, they provide pharmacological context for considering whether the need for rapid systemic exposure differs according to the site and severity of infection [4].

Within this context, our finding of no apparent incremental efficacy benefits from an initial loading dose, together with a numerically higher incidence of nephrotoxicity among patients receiving a loading dose, provides additional evidence relevant to the unresolved balance between systemic exposure and renal safety. Although the difference in nephrotoxicity was not statistically significant, the direction of the association raises the possibility that omission of the loading dose may be a reasonable strategy in selected patients with urinary tract infection, particularly when the anticipated benefit of rapidly increasing systemic exposure is limited. This finding should be interpreted cautiously, however, and prospective studies are needed to determine whether a non-loading-dose regimen can preserve efficacy while reducing renal toxicity.

Several limitations of this study warrant consideration. First, this was a retrospective, observational study conducted at a single regional tertiary hospital, which may limit the generalizability of our findings to other settings with different prescribing patterns or resistance profiles. Although we utilized rigorous statistical adjustment via inverse probability weighting (IPW) to minimize confounding by indication, the potential for residual confounding from unmeasured variables cannot be eliminated. In particular, loading-dose status was correlated with maintenance dosing intensity (Table 2), and because our IPW model adjusted only for baseline covariates, it could not fully equalize this post-baseline difference in cumulative drug exposure between groups. Second, our sample size was relatively modest (*n* = 123), which may have limited our statistical power to detect smaller differences in clinical outcomes or to reach statistical significance for the observed trend in nephrotoxicity risk. Third, due to the retrospective nature of the study, we were unable to measure direct urinary concentrations of CMS or active colistin, meaning our pharmacological explanations remain inferred based on established pharmacokinetic principles rather than direct localized measurements. Fourth, bacterial susceptibility to colistin was determined using a commercial broth microdilution system. While this aligns with global standards, potential variations in microbial minimum inhibitory concentrations (MICs) throughout the 7-year study period were not dynamically modeled. Fifth, we cannot fully exclude the possibility that some episodes classified as UTI represented asymptomatic bacteriuria or catheter-associated colonization rather than true infection, particularly given the high overall severity of illness in this cohort (mechanical ventilation in 57.7%, intensive care unit admission in approximately 40%, and septic shock in 39–47% of patients) and the identification of concurrent extra-urinary diagnoses in 20.3% of patients. Catheterization status and blood culture (bacteremia) results were not systematically recorded in our retrospective dataset and could therefore not be reported. Their absence limits our ability to confirm that the urinary tract, rather than a concurrent or alternative site, was the principal source of infection driving the outcomes studied, and this in turn limits confidence in the pharmacological argument in Section 3.2 and Section 3.3, which assumes infection confined to the urinary tract. We were similarly unable to reliably sub-classify infections as cystitis, pyelonephritis, obstructive UTI, or catheter-associated infection or to characterize source-control procedures performed. We flag these as priority variables for structured collection in future prospective studies. Sixth, although we classified UTI as complicated based on infection with a multidrug-resistant or extensively drug-resistant organism in hospitalized patients, we did not require or separately assess a structural or functional urinary tract abnormality as a criterion. This operational definition should be considered when comparing our findings to studies using anatomically based definitions of complicated UTI.

## 4. Methods

### 4.1. Study Design and Setting

This was a retrospective cohort study conducted at Nakornping Hospital, an 800-bed tertiary regional hospital in Chiang Mai, Northern Thailand, between 1 January 2015 and 30 June 2022. The study protocol was reviewed and approved by the Ethics Committee of Nakornping Hospital for Human Research (Study ID: 093/65). Given the retrospective, observational nature of the study and the use of routinely collected clinical data, the requirement for individual patient informed consent was waived by the ethics committee. To protect patient confidentiality, all data were de-identified prior to analysis, and each patient was assigned a unique study code for data collection, entry, and management. All procedures were carried out in accordance with the relevant institutional guidelines and applicable regulations governing research involving human participants.

### 4.2. Study Participants

Patients were eligible for inclusion if they were 18 years of age or older and had received intravenous colistin (as colistimethate sodium) for the treatment of a urinary tract infection (UTI) caused by a multidrug-resistant or extensively drug-resistant Gram-negative organism, confirmed by microbiological culture. Patients were categorized into two comparison groups according to whether colistin therapy was initiated with a loading dose or without a loading dose, as determined by the treating physician. Dosing, including any reduction for renal impairment, was determined empirically by the treating physician. No fixed institutional nomogram or creatinine-clearance-based dose-adjustment protocol was in use during the study period. Patients were excluded if colistin was administered for an indication other than UTI, if treatment duration was shorter than 48 h, if the patient was receiving renal replacement therapy at the time of treatment initiation, or if essential outcome data (e.g., follow-up urine culture, serum creatinine values, or vital status at 30 days) could not be retrieved from the medical record.

### 4.3. Definitions

UTI was defined as the presence of at least one urinary tract-related symptom (fever greater than 38 °C in patients 65 years of age or younger, suprapubic tenderness, costovertebral angle pain or tenderness, urinary frequency, urinary urgency, or dysuria), together with a positive urine culture growing no more than two microbial species, at least one of which was present at a colony count of ≥10^5^ CFU/mL. UTI was classified as complicated based on the presence of infection with a multidrug-resistant or extensively drug-resistant organism in a hospitalized patient, consistent with the prior literature classifying such infections as complicated regardless of underlying urinary tract structure. This study did not separately assess or require a structural or functional urinary tract abnormality, indwelling urinary catheter, or immunosuppression as a criterion for complicated UTI, and this operational definition should be distinguished from definitions requiring anatomical or functional abnormality. A loading dose was defined as an initial colistin dose administered at treatment initiation that exceeded the subsequent maintenance dose, given with the intent of more rapidly achieving therapeutic steady-state plasma concentrations.

The primary outcome was 30-day all-cause mortality, defined as death from any cause within 30 days after initiation of colistin therapy. Time origin was the date of colistin initiation. Patients who were alive and in follow-up at day 30 were censored at that date. Secondary outcomes included clinical response, microbiological response, and safety.

Clinical response was defined as resolution of the presenting signs and symptoms of UTI, or a sustained improvement in these signs and symptoms, by the end of colistin therapy. Clinical failure was defined as no improvement, persistence or worsening of at least one presenting symptom, or death attributable to the infection during treatment.

Microbiological response was defined as eradication of the previously isolated causative organism on a follow-up urine culture obtained at or after the end of antibiotic treatment. Microbiological failure was defined as persistence of the same organism on follow-up culture.

Safety was assessed based on the occurrence of nephrotoxicity, defined according to standard biochemical criteria, the Risk, Injury, Failure, Loss, and End-stage kidney disease (RIFLE) criteria. Specifically, colistin-associated nephrotoxicity was defined by the development of at least the ‘Risk’ stratum, characterized as an increase in serum creatinine of ≥1.5 times from the baseline value during or immediately after colistin therapy. Baseline demographic and clinical data, including age, sex, comorbidities (Charlson comorbidity index components, including chronic kidney disease and liver cirrhosis), baseline serum creatinine, and concomitant use of other nephrotoxic agents (e.g., vancomycin), were extracted from the medical records for use as covariates in the adjusted analysis.

### 4.4. Data Collection

Data were abstracted from electronic and paper medical records by trained investigators using a standardized case report form. Variables collected included patient demographics, underlying comorbidities and Charlson comorbidity index, indication and severity of infection, causative microorganism, colistin dosing regimen (loading versus non-loading), total and daily colistin dose, duration of therapy, concomitant antibiotic and nephrotoxic drug use, serum creatinine values at baseline and throughout treatment, follow-up urine culture results, and vital status at 30 days.

### 4.5. Statistical Analysis

Continuous variables were summarized as mean ± standard deviation or median with interquartile range, as appropriate, and compared between the loading-dose and non-loading-dose groups using the independent samples *t*-test or Mann–Whitney U test. Categorical variables were summarized as frequencies and percentages and compared using the chi-square test or Fisher’s exact test, as appropriate.

To account for baseline differences between the loading-dose (LD) and non-loading-dose (non-LD) groups, inverse probability weighting (IPW) based on the propensity score was applied to estimate the average treatment effect (ATE). Candidate covariates for inclusion in the propensity score model were first screened in univariate analyses (*p* < 0.20) and were also considered based on clinical relevance. The final propensity score was estimated using a multivariable logistic regression model including age, gender, chronic liver disease, chronic kidney disease, Charlson comorbidity index, baseline serum creatinine, and concomitant vancomycin use. Common support and the distribution of IPW weights were assessed visually using propensity score overlap plots and weight histograms. Covariate balance before and after weighting was evaluated using standardized mean differences (SMDs). IPW-adjusted analyses were conducted among patients with complete data for the propensity score covariates.

For 30-day all-cause mortality, Cox proportional hazards regression was used to estimate crude and IPW-adjusted hazard ratios (HRs). The proportional hazards assumption for the mortality model was assessed using Schoenfeld residuals (global *p* = 0.2262) and log–log survival plots. Clinical response and microbiological response were analyzed as binary outcomes using crude and IPW-weighted logistic regression, with results reported as odds ratios (ORs) and IPW-adjusted odds ratios (aORs). For nephrotoxicity defined by RIFLE criteria, competing risk analyses were performed using the Fine–Gray subdistribution hazards model, treating death as a competing event. Results were reported as subdistribution hazard ratios (sHRs) for crude analyses and IPW-adjusted subdistribution hazard ratios (asHRs) for weighted analyses.

A two-sided *p*-value < 0.05 was considered statistically significant. All analyses were performed using Stata software, version 14.0 (StataCorp, College Station, TX, USA).

### 4.6. Antimicrobial Susceptibility Testing

Bacterial isolates recovered from urine culture were identified at the Clinical Microbiology Division of Nakornping Hospital using standard culture techniques together with conventional biochemical identification methods. Susceptibility to a broad panel of clinically relevant antibiotics was initially assessed via the disk diffusion method, and results were interpreted according to the breakpoint criteria of the Clinical and Laboratory Standards Institute [22]. For the carbapenem class of antibiotics specifically, susceptibility and minimum inhibitory concentration (MIC) values were instead determined using the automated VITEK^®^ 2 system (bioMérieux, Marcy-l’Étoile, France), given the greater reliability of automated broth-based methods for these agents compared with disk diffusion. For colistin specifically, MIC values and susceptibility status were determined using the commercial broth microdilution system (Sensititre™, Thermo Fisher Scientific, Waltham, MA, USA), adhering to the joint guidelines of the Clinical and Laboratory Standards Institute (CLSI) and the European Committee on Antimicrobial Susceptibility Testing (EUCAST) [23] for polymyxin testing.

## 5. Conclusions

In conclusion, our study demonstrates that in patients with complicated urinary tract infections caused by multidrug-resistant or extensively drug-resistant Gram-negative pathogens, the administration of a colistin loading dose does not significantly reduce 30-day all-cause mortality or improve clinical response and microbiological eradication compared with a non-loading-dose regimen. A plausible pharmacological explanation is that colistin’s active form is already highly concentrated in the urinary tract through regular dosing, although this was not directly measured in our study. Nephrotoxicity rates were numerically lower in the non-loading-dose group, although this difference did not reach statistical significance (*p* = 0.110). For clinicians, these findings suggest that omitting the loading dose is a reasonable option for localized urinary infections, particularly in patients already at high risk for acute kidney injury, although the wide confidence intervals in this study of 123 patients preclude a definitive recommendation. Future multi-center randomized controlled trials with larger samples are needed to confirm these observations and to optimize safety-focused dosing strategies for polymyxins in localized infections.

## Figures and Tables

**Table 1:** Demographic and clinical characteristics of patients in loading-dose colistin and non-loading-dose groups.

Characteristic	Loading-Dose Colistin (*n* = 74)	Non-Loading Dose (*n* = 49)	*p*-Value
Sex (no. [%] of patients)			
Male	29 (39.19)	12 (24.49)	0.090
Female	45 (60.81)	37 (75.51)	
Age (years), mean ± SD	61.85 ± 17.24	66.97 ± 15.97	0.099
Duration of treatment, days, mean ± SD	8.98 ± 5.26	8.00 ± 4.49	0.284
Underlying diseases, *n* (%)			
Chronic obstructive pulmonary disease	7 (9.46)	4 (8.16)	0.805
Malignancy	21 (28.38)	14 (28.57)	0.981
Cardiovascular disease	19 (25.68)	15 (30.61)	0.549
Hypertension	33 (44.59)	24 (48.98)	0.633
Chronic liver disease	3 (4.05)	5 (10.20)	0.176
Chronic kidney disease	16 (21.62)	21 (42.86)	0.012
Diabetes mellitus	15 (20.27)	13 (26.53)	0.418
ICU status, *n* (%)	30 (40.54)	20 (40.82)	0.976
Septic shock, *n* (%)	35 (47.30)	19 (38.78)	0.351
Charlson Score, mean ± SD	2.35 ± 2.05	3.40 ± 2.47	0.011
Baseline SCr, mg/dL, mean SD	1.23 ± 1.34	1.88 ± 1.62	0.022
Type of nephrotoxic medications, *n* (%)			
Aminoglycosides	1 (1.35)	2 (4.08)	0.563
Diuretics	45 (60.81)	27 (55.10)	0.529
Amphotericin B	3 (4.05)	3 (6.12)	0.682
Vasopressor	35 (47.30)	19 (38.78)	0.351
Vancomycin	39 (52.70)	19 (38.78)	0.130
Length of hospital stay, days, mean ± SD	40.28 ± 34.07	37.79 ± 26.02	0.665
*A. baumannii*	51 (68.92)	30 (61.22)	0.318
*P. aeruginosa*	12 (16.22)	11 (22.45)	0.385
*K. pneumoniae*	3 (4.35)	2 (4.55)	1.000
*E.coli*	8 (10.81)	6 (12.24)	0.806

SCr, serum creatinine; SD, standard deviation.

**Table 2:** Colistin dosing characteristics in the loading-dose and non-loading-dose groups.

Characteristic	Loading-Dose Colistin (*n* = 74)	Non-Loading Dose (*n* = 49)	*p*-Value
Initial dose, mg CBA	300 (100%)	100–250 (median 150)	—
Duration of therapy, days, median (IQR)	7 (5–12)	7 (4–10)	0.665
Total cumulative dose, mg, median (IQR)	1925 (1143–3000)	1210 (675–2025)	<0.001
Average daily dose, mg/day, median (IQR)	283 (175–333)	150 (110–210)	<0.001

CBA, colistin base activity; IQR, interquartile range.

**Table 3:** Primary and secondary outcomes for patients who received a loading dose of colistin and a non-loading dose.

Outcome	Loading-Dose Colistin (*n* = 74)	Non-Loading Dose (*n* = 49)	*p*-Value
Primary outcome			
30-day all-cause mortality	26 (35.14)	20 (40.82)	0.524
Secondary outcomes			
Clinical response	56 (75.68)	35 (71.43)	0.599
Microbiological response	58 (78.38)	32 (65.31)	0.109
Safety			
Nephrotoxicity	36 (48.65)	19 (38.78)	0.281

**Table 4:** Adjusted clinical and safety outcomes between LD and non-LD colistin groups.

Outcome	LD Group (*n* = 74)	Non-LD Group (*n* = 49)	Crude (95% CI)	*p*-Value	Adjusted Metric ** (95% CI)	*p*-Value
Primary outcome						
30-day all-cause mortality *	26 (35.14%)	20 (40.82%)	HR: 0.76 (0.43–1.38)	0.370	aHR: 0.78 (0.43–1.42)	0.419
Secondary outcomes						
Clinical response	56 (75.68%)	35 (71.43%)	OR: 1.24 (0.55–2.81)	0.599	aOR: 1.48 (0.54–4.08) ^†^	0.445
Microbiological response	58 (78.38%)	32 (65.31%)	OR: 1.81 (0.74–4.42)	0.118	aOR: 2.10 (0.81–5.48) ^†^	0.128
Safety						
Nephrotoxicity (RIFLE criteria)	36 (48.65)	19 (38.78)	sHR: 1.57 (0.65–3.83) #	0.319	asHR: 2.23 (0.83–5.95) ^‡^	0.110

Footnotes: * aHR (adjusted hazard ratio) indicates the hazard of death. An HR < 1 indicates a lower risk of mortality in the LD group. ** Outcomes adjusted using inverse probability weighting (IPW) for the average treatment effect (ATE). Covariates in the propensity score model included: age, gender, liver cirrhosis, chronic kidney disease, Charlson comorbidity index, baseline serum creatinine, and concomitant vancomycin use. ^†^ Analyzed using IPW-weighted logistic regression for a binary outcome; results are reported as adjusted odds ratios (aORs). # Analyzed using the Fine–Gray unadjusted risk model. ^‡^ Analyzed using the Fine–Gray competing risk model (adjusted subdistribution hazard ratio), treating death as a competing risk for the onset of nephrotoxicity. The proportional hazards assumption was verified and met (global *p* = 0.2262).

## Data Availability

The datasets used and analyzed during the current study are available from the corresponding author upon reasonable request.
